# Development and description of New Nordic Diet scores across infancy and childhood in the Norwegian Mother, Father and Child Cohort Study (MoBa)

**DOI:** 10.1111/mcn.13150

**Published:** 2021-02-02

**Authors:** Neha Agnihotri, Elisabet Rudjord Hillesund, Elling Bere, Andrew Keith Wills, Anne Lise Brantsæter, Nina Cecilie Øverby

**Affiliations:** ^1^ Department of Nutrition and Public Health University of Agder Kristiansand Norway; ^2^ Department of Health and Inequalities and Centre for Evaluation of Public Health Measures Norwegian Institute of Public Health Oslo Norway; ^3^ Department of Sport Science and Physical Education University of Agder Kristiansand Norway; ^4^ Faculty of Health Sciences University of Bristol Bristol UK; ^5^ Division of Infection Control and Environmental Health Norwegian Institute of Public Health Oslo Norway

**Keywords:** child, diet scores, MBRN, MoBa, New Nordic Diet

## Abstract

In recent years, examining dietary patterns has become a more common way of investigating potential associations between diet and adverse health outcomes. The New Nordic Diet (NND) is a potentially healthy and sustainable dietary pattern characterized by foods that are locally available and traditionally consumed in the Nordic countries. The diet has been typically examined in adult populations, and less is known about compliance to the NND from infancy throughout childhood. In the current study, we therefore aimed to develop and describe child age‐specific NND scores. This study is based on the Norwegian Mother, Father and Child Cohort Study (MoBa) and uses data from the Medical Birth Registry of Norway (MBRN). We have previously developed a NND score for the maternal diet during pregnancy, and the development of the child diet scores was based on the rationale of this score. Food frequency data from *n* = 89 715 at child age 6 months, *n* = 76 432 at 18 months, *n* = 58 884 at 3 years, and *n* = 35 978 at 7 years were used to construct subscales in accordance with the maternal diet score. Subscales were composed of responses to a selection of food and drink items or other questions and were dichotomized by the median, yielding four age‐specific diet scores where the possible scoring ranged from 0 to 6 at 6 months and 3 years and from 0 to 9 at 18 months and 7 years. The developed scores will be used to examine associations with childhood overweight and cognitive and mental development in future studies.

**Key messages**

- What we eat is critical for human and planetary health; thus, identifying healthy and sustainable diets is important
- Dietary scores are used to explore diet–health associations. Few dietary scores are developed to assess child diet.
- We developed age‐specific child dietary scores to assess compliance with a healthy and potentially sustainable diet using data from the MoBa.
- The scores will be used in further studies to investigate their associations with childhood health outcomes.

## 1 INTRODUCTION

Within the last decades, there has been an increased focus on the relationship between dietary patterns and adverse health outcomes (Hu, 2002). Compared to examining health effects of single nutrients and foods, addressing dietary patterns may hold an advantage as it can encompass the complexity of different components in the diet and potential interaction between foods and nutrients (Waijers, Feskens, & Ocke, 2007). Compliance to a defined healthy dietary pattern is quantified through scores or indices and measures exposure to certain foods or nutrients (Kant, 2004).

Although health‐related associations with the Mediterranean diet have been thoroughly examined in various populations, the literature on other regional dietary patterns remains less extensive. The New Nordic Diet (NND) is a theoretically defined dietary pattern with foods that are traditionally consumed and locally available in the Nordic countries. In addition to encompassing foods that carry a Nordic identity and potential health‐promoting properties, the sustainability potential of the diet is also addressed in the NND (Bere & Brug, 2009; Mithril et al., 2012, 2013). The dietary composition consists of foods such as oats, rye, cabbages, root vegetables, apple, pears, berries, fish and game (Bere & Brug, 2009; Mithril et al., 2012).

Whereas employment of various Nordic diet scores and their relation to health‐associated outcomes has been more commonly examined in adult populations (Adamsson et al., 2011; Adamsson, Cederholm, Vessby, & Risérus, 2014; Enget Jensen et al., 2018; Hillesund, Bere, Haugen, & Øverby, 2014; Olsen et al., 2011; Poulsen, Crone, Astrup, & Larsen, 2015; Skreden et al., 2018), less is known regarding this dietary pattern in paediatric populations. In a systematic review from 2014, 80 diet quality indices were identified that were designed for use in paediatric populations (Marshall, Burrows, & Collins, 2014). None of these was from any Nordic countries.

A randomized controlled cross‐over trial, the OPUS Study, was conducted in Denmark, where the effects of serving school meals based on the NND to children aged 8–11 years were investigated (Andersen et al., 2015). As a result of increased intake of dietary fibre and protein and reduced intake of fat, the NND school meal resulted in improved blood pressure, insulin sensitivity and plasma triacylglycerol (Andersen et al., 2014). However, a small increase in waist circumference was also found, which was positively associated with potato consumption (Damsgaard et al., 2016). The OPUS intervention lasted for 6 months, and evidence of the health effects of early exposure and high compliance to the NND over time is as yet unclear.

Assessment of diet during infancy and in early childhood is important, as an adequate nutritional status during childhood is essential for proper child growth and development, with the first years of life being described as a crucial period for mental and physical development (Baidal et al., 2016; Pietrobelli, Agosti, & Group, 2017). Furthermore, the foundation of future dietary patterns has been suggested to be laid early in life (Robinson et al., 2007) and to track from infancy (Robinson et al., 2007) into childhood (Bjelland et al., 2013; Northstone & Emmett, 2008), as described by Lioret et al. (Lioret et al., 2015). Moreover, dietary behaviour in childhood may persist into adulthood (Hovdenak et al., 2019; Mikkilä, Räsänen, Raitakari, Pietinen, & Viikari, 2005), thus be early predictors of later health (Marshall et al., 2014).

We have previously developed a NND‐adherence diet score based on maternal dietary data in The Norwegian Mother, Father and Child Cohort Study (MoBa), aiming to embrace a combined health and sustainability perspective on a diet (Hillesund et al., 2014). The NND is a qualitative dietary pattern shown to be associated with better diet quality and higher adherence to dietary recommendations (Bjørnarå et al., 2016; Hillesund, Bere, et al., 2014).

Similar diet scores aiming to reflect NND compliance among children have, however, not yet been developed. In the current study, we aimed to develop and describe child diet scores reflecting NND compliance at child age 6 months, 18 months, 3 years, and 7 years, in line with rationale for the maternal NND score developed in MoBa.

## 2 MATERIALS AND METHODS

### 2.1 Study design and sample

The data used in this study were derived from MoBa, which is a population‐based pregnancy cohort study conducted by the Norwegian Institute of Public Health (Magnus et al., 2016). Participants were recruited from all over Norway from 1999 to 2008. The women consented to participation in 41% of the pregnancies. The cohort now includes 114 500 children, 95 200 mothers and 75 200 fathers. The current study is based on version 8 of the quality‐assured data files released for research in February 2014.

The mothers responded to three questionnaires during pregnancy and the father to one. Follow‐up of the participants has been conducted through questionnaires forwarded at a regular intervals, clinical sub‐studies and linkage to national health registries. Data collection is still ongoing.

Child diet at ages 6 months and 3 years was assessed with identical questions throughout the data collection; however, a few questionnaire items for child diet at 18 months and 7 years of age were changed during the data collection period. In the 18‐month questionnaire, response options differed to such a degree that inclusion of data from the first 5008 respondents was not possible to include in the construction of the diet score. In the 7‐year questionnaire, the assessment of consumption of Nordic fruits was hampered by grapes being grouped with apples and pears in some versions of the questionnaire. We used the available dietary data from all respondents at each time point except at 18 months for a reason described above.

### 2.2 METHODS

**FIGURE 1:**
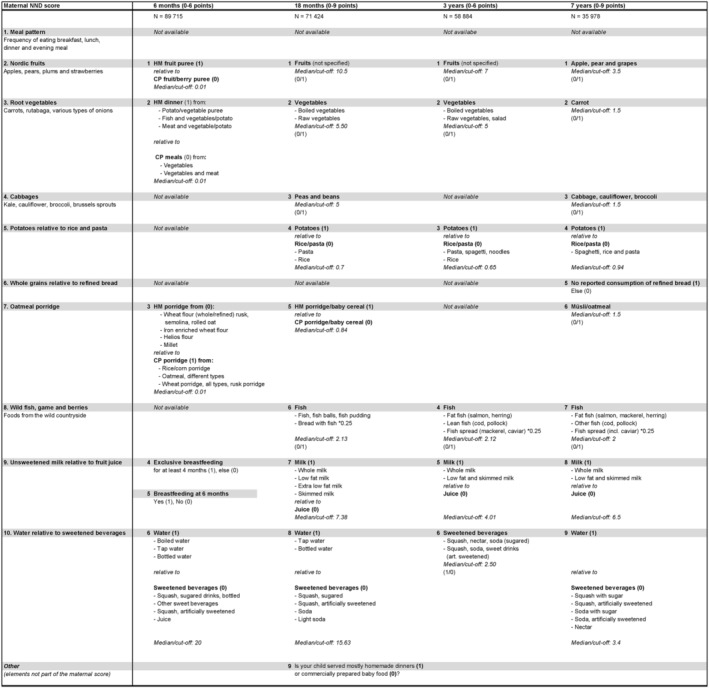
Details of the construction of the child New Nordic Diet (NND) scores based on the previously developed maternal NND score (Hillesund, Bere, et al., 2014). Abbreviations: CP = commercially prepared, HM = homemade. The table shows the items included in constructing the subscales of the child scores. Subscales of the maternal score are shown on the left, and corresponding child subscales at each age stage are shown accordingly. Coding/scoring of the subscales is in the brackets. The direction of scoring above or below the median is indicated by the following: (0/1 = *Weekly consumption above the median is given 1 point*; 1/0 = *Weekly consumption below the median is given 1 point*.) Deviations and differences in the scores are due to the limited dietary assessment in the child questionnaires from the Norwegian Mother, Father and Child Cohort Study

The dietary items included in the MoBa questionnaires, referred to as child food frequency questionnaires (FFQs) assessed the frequency of intake of breast milk, porridges, baby foods, dairy products, bread, fish, meat, fruits, vegetables, sweetened beverages and sweets and snacks, with the selection of questions adapted to child age in the respective questionnaires. The MoBa child FFQs did not cover the whole diet and have not been validated against other dietary assessment methods. However, a previous study in the same dataset showed fair to moderate tracking of fruit, vegetable and sugar‐sweetened beverage intakes from 18 months to 7 years in MoBa (Bjelland et al., 2013). The number of FFQ items included in the construction of each NND score is detailed below and in Figure 1.

The 6‐month questionnaire comprised 28 food and drink items, of which 23 were used to construct the 6‐month score. In addition, breastfeeding duration and frequency were assessed with two questions. Mothers ticked off whether the child was breastfed at each consecutive month between 0 and 6 months (seven categories) and reported frequency of breastfeeding with response alternatives ranging from ‘*never/seldom*’ to ‘*at least once a day*’. Exclusive breastfeeding duration, that is, for how long the infant received breast milk with no supplementary milk and/or solid food, was calculated from the question of duration of breastfeeding and/or other types of milk feeding and the question about the time of introduction to various types of solid food. For fruit puree, dinner and porridge, the questions differentiated between homemade and commercially produced foods.

At 18 months, the questionnaire food list comprised 39 food items, of which 23 were used in the construction of the diet score. Frequency of intake was assessed with seven response categories ranging from ‘*never*’ to ‘*five times or more a day*’ for drinks and from ‘*never*’ to ‘*three times a day or more*’. One question asked if the child was mostly served homemade dinners or commercially prepared baby food, with five response alternatives ranging from ‘*only homemade*’ to ‘*only commercially prepared*’.

In the questionnaire at 3 years, the questionnaire food list comprised 37 items, of which 14 were used in the construction of the score. Seven response options were available, ranging from ‘*seldom/less than once a week*’ to ‘*four times or more a day*’ for spreads, drinks, sweets and snacks and from ‘*once a month or less*’ to ‘*five times a week or more*’ for whole foods or dinner items.

At 7 years, the questionnaire food list comprised 47 food and drink items, of which 19 were used in the construction of the score. Frequency of intake was assessed with six response categories ranging from ‘*never/seldom*’ to ‘*once a day or more*’ for foods and from ‘*never/seldom*’ to ‘*four glasses a day or more*’ for drinks. All response options were recoded to reflect weekly consumption of a given food or drink item.

### 2.3 The rationale

We intended to develop child NND scores that could capture similar dietary aspects as the previously developed maternal NND score (Hillesund, Overby, et al., 2014), however, accepting that some of the dietary elements might not be available in the child questionnaires. The rationale of the maternal score was to include food items and dietary habits considered to be both healthy and possibly sustainable, by focussing on local foods with a potential Nordic identity, their tradition or importance as a food source in Nordic countries and their health potential in comparison to similar foods within the same food group (Hillesund, Overby, et al., 2014). Based on this, a score comprising 10 subscales was developed. The subscales of the maternal NND score in MoBa are presented below:

1. Meal pattern: combined frequency of eating breakfast, lunch, dinner and evening meal
2. Nordic fruits: frequency of eating apples, pears, plums and strawberries
3. Root vegetables: frequency of eating carrots, rutabaga and various types of onions
4. Cabbages: frequency of eating kale, cauliflower, broccoli and Brussels sprouts
5. Potatoes: frequency of eating potatoes relative to rice and pasta
6. Whole grain breads: frequency of consuming whole grain breads relative to refined breads
7. Oatmeal porridge: frequency of eating oatmeal porridge
8. Foods from the wild countryside: frequency of eating game, fish, seafood and native berries
9. Milk: frequency of consuming unsweetened milk relative to fruit juice
10. Water: frequency of consuming water relative to sweetened beverages.

### 2.4 Construction of the child diet scores

For each age, relevant diet‐related variables from the questionnaires were selected to construct subscales as similar as possible to the subscales in the maternal NND score. All respondents at each age assessment were included for development of the scores and in determining the subscale cutoffs for scoring (*n* = 89 715 at 6 months, *n* = 76 432 at 18 months, *n* = 58 884 at 3 years and *n* = 35 978 at 7 years). This was done to maximize data use and for representativeness.

For any reported consumption of spreads included in a subscale, the frequency was multiplied by 0.25 to down weight spreads to a quarter of whole foods given that spreads are generally eaten more frequently, but in smaller amounts. In the subscales where consumption of a food group/beverage was compared to the consumption of another food group/beverage (e.g., water relative to sweet beverages), a formula was used to get a relative measure of consumption. To avoid zero in the denominator in cases where unhealthy foods were reported to be consumed never or seldom (0), 0.1 was added in the denominator as presented in the formula below:

$$\frac{\left(\text{Healthy food}\;1+\text{Healthy food}\;2+\ldots \right)\;}{\left(\left(\text{Unhealthy food}\;1+\text{Unhealthy food}\;2+\ldots \right)+0.1\right)}.$$

Missing was defined as having incomplete data on all food items included in the construction of each child diet score. This was the case for *n* = 284 (0.3%) at 6 months, *n* = 257 (0.4%) at 18 months, *n* = 1076 (1.8%) at 3 years and *n* = 343 (1.0%) at 7 years. For the remaining missing food items, an assumption of null intake was made in accordance with recommendations by Cade, Thompson, Burley, and Warm, (2002). These items were recorded to 0 (*never*/*seldom*) to avoid losing all dietary information for respondents with incomplete data for a given item.

Most subscales were dichotomized by the median (frequency of weekly consumption) [31] and coded to give either 0 or 1 point, where receiving 1 point acknowledged a healthier food choice or consumption above the median. Three subscales (still breastfeeding at 6 months, offering homemade vs. commercial baby foods at 18 months, and no reported consumption of refined bread at 7 years) were based on single questionnaire responses only and were scored according to having the behaviour or not. The sum of the subscales was further computed to yield a continuous NND child score.

### 2.5 Characteristics according to NND scoring

Child gender and maternal age at delivery were derived from the Medical Birth Registry of Norway (MBRN), a national health registry containing information about all births in Norway. Parental educational attainment, measured by highest completed educational level, was derived from the MoBa baseline questionnaire (Q1). We collapsed education categories into a variable with three categories corresponding to ≤12, 13–16, and ≥17 years of education. We further grouped participants as having low, medium or high NND score at each respective stage, with cutoffs chosen to yield approximately equal proportion in high and low NND categories (see Figure 1 for details). We present maternal age, parental education and child gender across categories of low, medium and high NND scoring at each time point. The Statistical Package for the Social Sciences (IBM SPSS Statistics, version 24.0) was used to analyse and handle the data.

### 2.6 Ethical considerations

The establishment of MoBa and initial data collection was based on a licence from the Norwegian Data Protection Agency and approval from The Regional Committees for Medical and Health Research Ethics. The MoBa cohort is now based on regulations related to the Norwegian Health Registry Act. The current study was approved by The Regional Committees for Medical and Health Research Ethics (Reference 2019/339).

## 3 RESULTS

Age‐specific NND scores were developed according to the presented rationale for ages 6 and 18 months and 3 and 7 years. Due to variations in the collected dietary data in the respective questionnaires, each diet score was built from available dietary components resembling as far as possible the ones included in the maternal score. Figure 1 shows details about the dietary variables included in each subscale, cutoffs for scoring and how each subscale was scored.

### 3.1 NND score at 6 months

In the NND score at 6 months, we included breastfeeding and homemade versus commercially produced food as elements of a healthy and sustainable diet. From the perspective of sustainability, breastfeeding is recommended as it leaves less environmental burden compared to feeding the infant with formula milk or other breast milk substitutes (Nasjonalt råd for ernæring, 11/2017). Furthermore, the wide range of health benefits of breastfeeding is well known (Lagström, Lande, & Thorsdottir, 2013). Norwegian health authorities recommend exclusive breastfeeding for at least 4 months and for 6 months if possible for mother and child, which deviates somewhat from World Health Organization (WHO) recommendations of 6 months (Helsedirektoratet, 2016). A very low proportion of mothers reported exclusive breastfeeding until child age 6 months. In the 6‐month score, infants were therefore given one point for being exclusively breastfed for at least 4 months. Another point was given for still being breastfed when completing the 6‐month questionnaire (mean age for the sample: 6.4 months; 196 days, SD: 15.1 days). Three out of six subscales in this score address homemade relative to commercially prepared food. The six subscales are presented below.

1. Consuming homemade fruit puree more frequently than commercially prepared fruit puree
2. Consuming homemade dinners more frequently than commercially prepared dinners
3. Consuming homemade porridge more frequently than commercially prepared porridge
4. Being exclusively breastfed for at least 4 months
5. Still being breastfed at the time of responding to the 6‐month questionnaire
6. Drinking water far more frequently than sweetened beverages (see Figure 1).

The subscales were summarized into a 6‐month NND score ranging from 0 to 6 points, with median score 3 (interquartile range, IQR 2–4).

### 3.2 NND score at 18 months

The score at 18 months consists of nine subscales (listed below), with one subscale assessing what degree homemade porridge was served compared to commercially prepared porridge and another addressing consumption of homemade dinners relative to commercially prepared baby dinner. Three subscales are in line with subscales in the maternal NND score, addressing consumption of potatoes, milk and water. One subscale addresses the consumption of peas and beans, and two of the subscales measure fruit and vegetable consumption, although not specifically of Nordic origin. The nine subscales are presented below with their respective cutoff for scoring. See Figure 1 for detailed information on food items included in each subscale.

1. Fruits: eating fruits more than 10.5 times a week
2. Vegetables: eating vegetables more than 5.5 times a week
3. Peas and beans: eating peas and beans more than five times a week
4. Potatoes: eating more potatoes relative to rice and pasta
5. Porridge: eating more homemade porridge/baby cereal relative to commercially prepared porridge/baby cereal
6. Fish: eating fish more than 2.13 times a week
7. Milk: drinking more milk relative to fruit juice
8. Water: drinking more water relative to sweetened beverages
9. Homemade food: eating more homemade dinners relative to commercially prepared baby food.

Summarizing the subscales, the NND diet score at 18 months could take values from 0 to 9 points, with median score 4 (IQR 3–5).

### 3.3 NND score at 3 years

The score at 3 years consists of six subscales (listed below). The questions regarding child diet in the questionnaire at 3 years were not as extensive as in the questionnaires at 6 months, 18 months and 7 years. There was no question addressing water intake, and subscale 6 in this score was therefore constructed to score respondents with consumption of sweetened beverages below the median and not as a relative measure as was done in the other scores. The item estimating whole‐grain bread consumption was unfortunately poorly constructed and yielded large amounts of missing data (12.7%); we therefore chose to exclude the variable. There was also no information on specific fruits or vegetables. The six subscales are presented below, with the cutoff for scoring in each subscale. See Figure 1 for detailed information on subscale construction.

1. Fruits: eating fruits more than seven times a week
2. Vegetables: eating vegetables or salad more than five times a week
3. Potatoes: eating more potatoes relative to rice and pasta
4. Fish: eating fish more than 2.12 times a week
5. Milk: drinking more milk relative to fruit juice
6. Sweetened beverages: drinking sweetened beverages less than 2.5 times a week.

After summarizing the subscales, the NND score at 3 years ranged from 0 to 6 points, with median score 3 (IQR 2–4).

### 3.4 NND score at 7 years

The score at 7 years consists of nine subscales (listed below). The 7‐year questionnaire provided the most detailed information of child diet, and this score is thus the most similar to the maternal NND score (Figure 1). The item assessing bread consumption had many missing due to how the question was constructed. Mothers reported how many slices of bread the child ate daily, distinguishing between white bread, semi‐white bread, whole grain bread and crispbread. We collapsed the white bread category with semi‐white bread and chose to score respondents with no consumption of white/semi‐white bread. The item that was included for measuring the consumption of potential local fruits in subscale 1 was a grouped item where grapes were also included with apples and pears. The nine subscales are presented below with cutoff for scoring in each subscale. See Figure 1 for detailed information on subscale construction.

1. Local fruits: eating apple, pear and grapes more than 3.5 times a week
2. Root vegetables: eating carrots more than 1.5 times a week
3. Cabbages: eating kale, cauliflower and broccoli more than 1.5 times a week
4. Potatoes: eating more potatoes relative to rice and pasta
5. Whole grain bread: reporting no consumption of white bread
6. Oatmeal: eating muesli or oatmeal more than 1.5 times a week
7. Fish: eating fish more than two times a week
8. Milk: drinking more milk relative to fruit juice (Figure 1)
9. Water: drinking more water relative to sweetened beverages (Figure 1).

After summarizing the subscales, the NND score at 7 years score ranged from 0 to 9 points, with median score 4 (IQR 3–6).

### 3.5 Parental and child characteristics according to NND scoring

To assess potential patterning by parental characteristics, we compared parental educational attainment, maternal age and child gender across low, medium and high NND scoring at each dietary assessment time point (Table 1). Compared to low and medium NND scores, high NND score at any assessment time point was associated with a substantially higher proportion of both mothers and fathers with high educational attainment pre‐pregnancy. High NND score was also associated with slightly higher maternal age at delivery except at 7 years and a slightly higher proportion of girls at 6 months and 3 years (Table 1).

**TABLE 1:** Parental and child characteristics according to NND scoring (low, medium and high score)

Characteristics	NND scoring^a^		
	Low	Medium	High
6‐month score			
Scoring^b^	0–2	3–4	5+
% of participants in each category	28.1	54	17.8
Child gender (% girls)	47.6	48.9	50.3
Maternal education^c^			
17 + years (%)	16.8	27.0	35.5
Paternal education^c^			
17 + years (%)	16.3	25.1	31.6
Maternal age^d^	29.6 (4.8)	30.4 (4.4)	30.7 (4.3)
18‐month score			
Scoring	0–3	4–5	6+
% of participants in each category	33.7	43.1	23.2
Child gender (% girls)	48.0	49.1	49.6
Maternal education^c^			
17 + years (%)	21.7	28.0	32.8
Paternal education^c^			
17 + years (%)	20.4	25.3	30.1
Maternal age^d^	30.0 (4.5)	30.5 (4.4)	30.7 (4.4)
3‐year score			
Scoring	0–1	2–3	4+
% of participants in each category	19.5	49.5	31.0
Child gender (% girls)	45.5	49.1	50.4
Maternal education^c^			
17 + years (%)	22.5	26.4	32.5
Paternal education^c^			
17 + years (%)	20.4	24.3	28.7
Maternal age^d^	29.9 (4.5)	30.4 (4.4)	30.8 (4.4)
7‐year score			
Scoring	0–3	4–5	6+
% of participants in each category	28.2	43.4	28.4
Child gender (% girls)	48.2	48.7	48.7
Maternal education^c^			
17 + years (%)	19.3	24.2	28.5
Paternal education^c^			
17 + years (%)	17.9	23.3	27.8
Maternal age^d^	30.3 (4.5)	30.4 (4.4)	30.6 (4.4)

^a^ NND: New Nordic Diet.

^b^ Defined NND scoring for low, medium and high for 6‐month score, 18‐month score, 3‐year score and 7‐year score.

^c^ Percentage of mothers and fathers with the highest level of education pre‐pregnancy, 17 years or more.

^d^ Maternal age at delivery, mean (SD).

## 4 DISCUSSION

We developed four child diet scores to capture a healthy and potentially sustainable diet at various stages throughout childhood, largely in line with the previously developed NND score for pregnancy diet in MoBa (Hillesund, Bere, et al., 2014). To our knowledge, these are the first scores that have been developed to assess adherence to a healthy and potentially sustainable diet in Norwegian children across infancy and into childhood. Despite some variation across questionnaires in child diet questions, we aimed to keep each child score as true to the original maternal score as possible. This was, however, challenging as not all elements were available at the different measure points. There were also limited data regarding food variety in the child questionnaires. In addition, options were aggregated including both Nordic and non‐Nordic food (i.e., grapes) reducing the applicability to reflect a potentially sustainable diet.

Despite these limitations, most of the subscales are largely in line with the corresponding maternal NND subscales, with an exemption of the score at 6 months, where the only similar subscale was the relative measure of water versus sweetened beverages. In this score, we chose to value breastfeeding as an obvious healthy and sustainable feeding practice (Myr, 2008) and homemade versus commercially produced baby foods as a potentially healthy and sustainable dietary choice. Homemade food was similarly valued in the 18‐month score. According to a recent narrative review by Maslin and Venter (Maslin & Venter, 2017), there is a paucity on studies comparing the nutritional content and composition in homemade food with commercially prepared infant food. There is no clear consensus regarding either preparation method being superior to the other in terms of, for example, micronutrient content. A randomized controlled intervention targeting parents of infants, aiming to enable parents to prepare homemade baby food, did show that serving the infant homemade porridge regularly was associated with higher high‐density lipoprotein (HDL) concentration at 24 months of age (Overby, Hernes, & Haugen, 2017). Still, as commercially produced porridges are fortified with iron and other nutrients, and at present are low in sugar (changed after early 2000), this may also be a healthy alternative. However, there is reason to believe that commercially produced baby foods could have a larger environmental impact than baby foods prepared at home, as, for example, more servings can be prepared in bulks at home with less packaging per meal. On the other hand, food waste could also be higher.

Meal frequency and consumption of foods from the wild countryside were not assessed in the child questionnaires; however, the latter subscale was substituted by scoring a weekly consumption of fish above the median. Although not fully in line with the maternal score, a plant‐based diet with more calories from the sea is encouraged in the original NND (Mithril et al., 2012).

Whereas the score at 6 months and partly at 18 months may capture sustainability aspects to a larger degree, the score at 3 years may be more reflective of a generally healthy diet. At 7 years, characteristic Nordic fruits and vegetables, and muesli and oatmeal are incorporated in the subscales, resulting in a score comprising of more Nordic elements. It should be noted that the scores measure health and sustainability aspects of diet differently at the four time points due to how they are composed.

The developed diet scores include many of the food groups that are commonly consumed in a Nordic diet, and their relation to health outcomes was examined in a systematic review from 2013 (Åkesson et al., 2013). The review included potatoes, berries, whole grains, dairy products and red and processed meat. No conclusions could be drawn regarding the health effects of potatoes and berries, but there was evidence of a probable protective association between whole‐grain intake and type 2 diabetes and coronary vascular disease risk. Potatoes are, however, regarded as a healthier option to rice and pasta because of their nutritional content, if not conventionally processed or fried (Camire, Kubow, & Donnelly, 2009) and are generally a more sustainable food choice in the Nordic countries (Hess, Chatterton, Daccache, & Williams, 2016).

Regarding dairy intake, there is suggestive evidence of a protective effect on type 2 diabetes and also an increased risk of prostate cancer (Åkesson et al., 2013). The NND guidelines by Mithril et al. does not elaborate on dairy products, other than that the diet follows the Danish food‐based guidelines (Mithril et al., 2013). Among dairy products in the current developed scores, only milk consumption as a relative measure to consuming juice was included, in line with the rationale behind the maternal NND score, which was to recognize a healthier option within the same food group (milk vs. juice) (Hillesund, Overby, et al., 2014). Game is the only meat element included in the maternal NND score; however, it was not addressed in the child dietary questionnaires and the scores do not assess meat intake.

It has been reported that the Mediterranean diet and the NND have almost similar GHG emissions and both are regarded as environmentally friendly diets (Ulaszewska, Luzzani, Pignatelli, & Capri, 2017). This could likely be transferable to the developed child diet scores, although more knowledge on the sustainability potential of homemade food compared to commercially prepared baby meals is needed.

### 4.1 Strengths and limitations

The data used for the development of the scores were derived from a large, prospective, population‐based cohort study conducted in Norway, which is a major strength of the study. There are, however, limitations that need to be addressed. The participation rate in MoBa cohort study has been decreasing over the years, and the mothers who initially chose to participate in MoBa were mostly older, cohabitating, non‐smokers and frequent users of multivitamin and folic acid supplement (Nilsen et al., 2009). This could have implications for the distribution of dietary intake in the MoBa sample and for the cutoffs that were derived from the data. Using the median to assess cutoff values in diet scores has been frequently debated, despite being widely used (Waijers et al., 2007). The method implies arbitrary cutoffs that may yield different results in populations with different diets. On the other hand, it does allow for a differentiation within a given sample or population (Waijers et al., 2007).

The questions regarding child diet were less detailed than those of maternal diet in MoBa, and not from validated FFQs. This may impact the validity of the child diet scores and their ability to capture the concept of the NND. In addition, the lack of detail may reduce the possibilities of identifying associations with health outcomes. However, we believe that the indices discriminate individuals according to beneficial dietary practices and are therefore valuable specifically in a dataset like MoBa with a high number of participants. Underpinning the value, our scores are comparable with the few other dietary scores developed in this age group. A British study from (Robinson et al., 2007) used principal component analysis of FFQ data to derive dietary patterns of infants at 6 months of age and identified a pattern characterized by a high frequency of consumption of vegetables, fruit, meat and fish and home‐prepared foods, a greater consumption of breast milk and a lower intake of commercial baby foods in jars and formula milk. The authors suggested that this pattern complied with the infant guidelines in the United Kingdom. Mothers of infants with high scores had higher educational attainment and a higher prudent diet score. Further, the score at 6 months deviates substantially from the original idea of the NND score due to natural limitations regarding what 6‐month‐olds eat and because the dietary information was far less specific at this age. However, as previously asserted, breastfeeding should be regarded as a part of the NND. Despite that this score does not truly reflect the components of the maternal NND score, some aspects of diet quality and sustainability may still be covered by acknowledging breastfeeding and meals prepared at home. A limitation also relates to the assumed superior health effects of homemade porridge compared to commercially prepared porridge. The composition of commercially prepared porridges has changed since the start of the MoBa, and today, they may be just as healthy as homemade porridge.

The strong educational patterning across diet scoring may introduce bias in future analyses of diet–health associations and should be properly addressed and controlled. Another limitation is the lack of possibility to control for energy intake in the child datasets, as a high score in individual participants could partly reflect higher food intake in addition to better diet quality (Hillesund, Overby, et al., 2014; Roswall et al., 2015). The diet scores do not capture meat intake, which is another shortcoming regarding sustainability. By including foods with a potential Nordic identity and a possibility for them to be locally grown, we attempted to include sustainability as an aspect of the scores. It is, however, impossible to know if true sustainability is addressed, as sustainability is a complex subject with many dimensions (Burlingame & Dernini, 2012).

As a result of assessing the NND through age‐specific diet scores based on limited food variety in the dietary assessments, we may not have been able to fully capture the true exposure of the NND in the study population. It should be emphasized that the scores are not intended to reflect the healthiest diet possible.

## 5 CONCLUSION

Child diet scores were developed in MoBa to measure compliance to the NND at child age 6 and 18 months and 3 and 7 years. Albeit the scores could not fully resemble the former developed maternal NND score in MoBa, aspects of a potentially healthy, local and sustainable diet are still maintained, although differently and to a varying degree at each age. Healthy and potentially sustainable eating in children should be addressed in more recent population studies as there has been increased attention in the general population on sustainability, regional foods and healthy eating. The developed scores will be used in further studies to investigate their associations with child and maternal characteristics, childhood obesity and cognitive and mental development within the MoBa.

## CONFLICTS OF INTEREST

The authors declare that they have no conflict of interest.

## CONTRIBUTIONS

NCØ, NA, EB and ERH contributed to the study conception and design. Data collection and data curation was managed by ALB. Material preparation and analysis were performed by NA, NCØ, AW, EB and ERH. The first draft of the manuscript was written by NA, and all authors commented on and revised following versions of the manuscript. All authors have read and accepted the final version of the manuscript.

## Data Availability

The consent given by the participants does not open for storage of data on an individual level in repositories or journals. Researchers who want access to data sets for replication should submit an application to datatilgang@fhi.no. Access to data sets requires approval from The Regional Committee for Medical and Health Research Ethics in Norway and an agreement with MoBa.
